# Characterising root trait variability in chickpea (*Cicer arietinum* L.) germplasm

**DOI:** 10.1093/jxb/erw368

**Published:** 2016-10-06

**Authors:** Yinglong Chen, Michel Edmond Ghanem, Kadambot HM Siddique

**Affiliations:** 1 The UWA Institute of Agriculture, The University of Western Australia, LB 5005, Perth WA 6001, Australia; 2 The State Key Laboratory of Soil Erosion and Dryland Farming on the Loess Plateau, Institute of Soil and Water Conservation, Northwest A&F University, and Chinese Academy of Sciences, Yangling, Shaanxi 712100, China; 3 International Centre for Agricultural Research in the Dry Areas, North-Africa Platform, Rabat, Morocco

**Keywords:** Adaptation, chickpea, *Cicer arietinum*, crop phenotyping, root system architecture, root trait variability.

## Abstract

Chickpea (*Cicer arietinum* L.) is an important grain legume crop but its sustainable production is challenged by predicted climate changes, which are likely to increase production limitations and uncertainty in yields. Characterising the variability in root architectural traits in a core collection of chickpea germplasm will provide the basis for breeding new germplasm with suitable root traits for the efficient acquisition of soil resources and adaptation to drought and other abiotic stresses. This study used a semi-hydroponic phenotyping system for assessing root trait variability across 270 chickpea genotypes. The genotypes exhibited large variation in rooting patterns and branching manner. Thirty root-related traits were characterised, 17 of which had coefficients of variation ≥0.3 among genotypes and were selected for further examination. The Pearson correlation matrix showed a strong correlation among most of the selected traits (*P*≤0.05). Principal component analysis revealed three principal components with eigenvalues >1 capturing 81.5% of the total variation. An agglomerative hierarchical clustering analysis, based on root trait variation, identified three genotype homogeneous groups (rescaled distance of 15) and 16 sub-groups (rescaled distance of 5). The chickpea genotypes characterised in this study with vastly different root properties could be used for further studies in glasshouses and field trials, and for molecular marker studies, gene mapping, and modelling simulations, ultimately aimed at breeding germplasm with root traits for improved adaptation to drought and other specific environments.

## Highlight

Large variation in root system architecture in chickpea germplasm was identified across 270 genotypes, providing information that can be used in developing genotypes with root traits for improved adaptation to specific environments.

## Introduction

Chickpea (*Cicer arietinum* L.) is the third most important pulse crop and is grown on about 12 million ha of land from temperate to sub-tropical regions of the world, with some 72% of world production from South Asian countries (Siddique *et al.*, 2012; FAOSTAT, 2014; Foyer *et al.*, 2016). The cultivation of chickpea covers some major agro-ecological environments, including stored soil moisture systems in South Asia, in-season rainfall in Mediterranean regions, alkaline sands in North India, alluvial soils in northwest India and Nepal, and lower water-holding-capacity soils in southern Australia (Siddique and Sedgley, 1986; Berger and Turner, 2007; Kashiwagi *et al.*, 2015). Sustainable production of chickpea is challenged by climate changes, which are likely to increase production limitations and uncertainty of yields in the future. Edaphic stresses, such as drought and low-fertility soils, are the main factors restricting the production of chickpea as well as other major crops in many countries. Globally, drought stress reduces chickpea production by an estimated 33%, of which approximately 19% could potentially be recovered through genetic improvement efforts (Subbarao *et al.*, 1995; Varshney *et al.*, 2009; Farooq *et al.*, 2016).

Modification to root system architecture may improve desirable agronomic traits such as yield, drought tolerance, and resistance to nutrient deficiencies (Tuberosa *et al.*, 2002; Beebe *et al.*, 2006; Ghanem *et al.*, 2011). However, wide-scale use of root-related genetic information in breeding is hampered by relatively small mapping populations and inaccurate phenotyping (de Dorlodot *et al.*, 2007). Hence, accurate phenotyping and characterisation of root-related traits is important for translating recent physiological and genetic advances into an understanding of the role of root systems in improving yield and productivity (especially in drying environments). Selecting and breeding cultivars with root systems that use water and nutrients efficiently is an important strategy to increase crop adaptation to edaphic stress (Siddique *et al.*, 2001).

A core collection of chickpea genotypes has been established and contains a significant proportion of the world’s genetic resources for this species. This resource forms a broad genetic basis for phenotyping genotypic variation and genetic diversity in root-related traits for future improvements in breeding programs. Characterising genotypic variability in this core collection of chickpea is critical to identify genotypes with optimal root traits efficient in water and nutrient acquisition.

The objective of this study was to characterise genotypic variability in root traits in the chickpea collection using a novel semi-hydroponic system recently used for root studies in narrow-leafed lupin (*Lupinus angustifolius* L.) (Chen *et al.*, 2011*a*, *b*, 2012, 2016), wheat (*Triticum aestivum* L.), and barley (*Hordeum vulgare* L.) (Yinglong Chen, unpublished data). This study provides detailed descriptions of the phenotypic variability and genetic diversity in root architectural traits in the chickpea core collection.

## Materials and methods

### Plant material and root phenotyping system

A collection of chickpea (*Cicer arietinum* L.) genotypes from 29 countries consisting of 270 genotypes (including two wild relatives of chickpea *C. echinospermum*) – primarily landraces with a few advanced cultivars and breeding materials – was used in this study. Seeds of the collection were provided by the International Crop Research Institute for the Semi-Arid Tropics and were then multiplied in a field station in Perth, Western Australia, prior to the experiment. The genotypes are listed in Supplementary Table S1 at *JXB* online, with information on country of origin and seed type. The climatic and eco-geographical data for each genotype together with genomic data for almost all genotypes tested in this study may be obtained from a number of sources, including the International Crop Research Institute for the Semi-Arid Tropics (http://www.icrisat.org), the National Bureau of Plant Genetic Resources (http://www.nbpgr.ernet.in), the International Centre for Agricultural Research in Dry Areas (http://www.icarda.cgiar.org), and the National Plant Germplasm System (http://www.ars-grin.gov/npgs/index.html) (Foyer *et al.*, 2016).

Root phenotyping was carried out using a novel semi-hydroponic phenotyping system (Chen *et al.*, 2011*a*) in a randomised block design with three biological replicates. Three replicate plants of each genotype were assigned to three separate bins. A total of 27 bins with nine bins in each replicate were established. Each bin system accommodated 32 plants with two plants per growth unit. Buffer plants were added when required to ensure that equal numbers of plants (32) were allocated to each bin. The phenotyping system has been previously described by Chen *et al.* (2011*a*). Each bin was filled with 30 l of solution containing (μM): K (1220), P (20), S (1802), Ca (600), Mg (200), Cu (0.2), Zn (0.75), Mn (0.75), B (5), Co (0.2), Na (0.06), Mo (0.03), Fe (20), and N (1000). The plant growth units maintained moisture via an automatic pumping system in each bin. The pH of the nutrient solution was monitored weekly, and the solution was renewed once every two weeks.

### Plant growth conditions and measurements

The experiment was undertaken in a temperature-controlled glasshouse at The University of Western Australia, Perth (31°58′ S, 115°49′ E). The average daily temperature was 22/16 °C (day/night), and the midday maximum photosynthetic photon flux density was 1742 μmol photons m^−2^ s^−1^ during the experimental period. Seeds were surface-sterilised and germinated in washed river sand. Emerged plants (about 7 d after sowing) were carefully transplanted into the growth pouches. Plants were harvested 35 d after sowing. Termination of the experiment for root growth assessment was determined when the taproots of the larger genotypes reached the bottom of the bin system (approx. 100cm depth). This was also based on our observations on a number of crop species (e.g. narrow-leafed lupin, wheat, and barley) using the same phenotyping platform. We found that plants grown for 4 to 6 weeks showed maximum root trait diversity among the genotypes tested.

At harvest, shoot height and leaf branch number per plant were measured. Root systems were photographed using a portable photographic system and taproot lengths were measured manually. After photographing, subsamples of roots were collected for morphological and architectural measurements by cutting the root system into 20-cm sections starting from the base. Shoots and roots were then dried in an air-forced oven at 70 °C for 72h and weighed to determine shoot and root dry weights. Root subsamples were optically scanned before drying (see below).

### Root scanning and image analysis

Root subsamples were scanned in greyscale at 300 dpi using a desktop scanner (Epson Perfection V700, Long Beach, CA, USA). Root images were analysed using WinRHIZO Pro software (v2009, Regent Instruments, Montreal, QC, Canada). The debris removal filter was set to discount objects less than 1cm^2^ with a length/width ratio less than 10. The roots were partitioned into 15 diameter classes at 0.15mm intervals and root length for each root diameter class was computed.

### Root-related traits

Root growth rate was calculated based on taproot length increments for the growth period (35 d). Data for various root traits, such as total root length, root surface area, root volume, average root diameter, and diameter class length (DCL, root length within a diameter class) were generated in WinRHIZO from images for each root section. The number of branches (primarily first-order) in the first root section was counted manually from the images. The parameters listed below were calculated based on observed and/or computed data.

Branch density (BD) − number of branches/taproot length.

Branch intensity (BI) − number of branches/root length.

Root tissue density (RTD) − root mass/root volume.

Root mass ratio (RMR) − root mass/total mass.

Root-to-shoot mass ratio (RMR) − root mass/shoot mass.

Specific root length (SRL) − root length/root mass.

Relative diameter class length (rDCL) − DCL/root length, yielding a proportion of root length to normalise disparity between plants of different sizes.

Root trait data in the upper 0–20cm (considered the ‘topsoil’ section) were compared with the rest of the root system (considered the ‘subsoil’ section). Descriptions and abbreviations of the 30 root traits and three shoot-related traits, i.e. shoot height (SH), leaf branch number (LBN), and shoot mass (SM), are presented in Table 1.

**Table 1.** Description of measured traits in a chickpea core collection grown in a novel semi-hydroponic phenotyping system

Trait	Abbreviation	Description	Units
Taproot length zone1	TRL_z1	Taproot length at branching zone (z1)	cm
Taproot length zone2	TRL_z2	Taproot length at non-branching zone (z2)	cm
Taproot length	TRL	Taproot length (i.e. root depth, z1+z2)	cm
Total root length	RL	Total root length per plant	cm
Total branch length	BL	Total root branch length	cm
Total branch number	BN	Total branch number	number per root
Average branch length	ABL	Branch length divided by branch number	cm per branch
Total root area	RA	Root surface area	cm^2^
Total root volume	RV	Root volume	cm^3^
Root diameter	RD	Average root diameter	mm
Specific root length	SRL	Root length over root dry mass	m g^–1^ dry mass
Branch length over taproot	BLR_tap	Branch length divided by taproot length	m m^–1^ taproot
Branch density	BD	Branch number divided by taproot length	cm^–1^ taproot
Branch intensity	BI	Branch number divided by total root length	cm^–1^ root
Root tissue density	RTD	Root dry mass divided by root volume	mg cm^–3^
Topsoil root length	RL_top	Root length in section 1 (s1, 0–20cm)	cm
Topsoil branch length	BL_top	Branch length in section 1 (s1, 0–20cm)	cm
Topsoil root diameter	RD_top	Average root diameter in section 1 (s1, 0–20cm)	mm
Root length s2	RL_s2	Root length in section 2 (s2, 20–40cm)	cm
Root diameter s2	RD_s2	Average root diameter in section 2 (s2, 20–40cm)	mm
Root length s3	RL_s3	Root length in section 3 (s3, 40cm and beyond)	cm
Root diameter s3	RD_s3	Root diameter in section 3 (s3, 40cm and beyond)	mm
Subsoil root length	RL_sub	Root length in subsoil (s2 and s3, 20cm and beyond)	cm
Subsoil branch length	BL_sub	Branch length in subsoil (s2 and s3, 20cm and beyond)	cm
Subsoil root diameter	RD_sub	Root diameter in subsoil (s2 and s3, 20cm and beyond)	mm
Root length ratio	RLR_top/sub	Topsoil root length divided by subsoil root length	
Branch length ratio	BLR_top/sub	Topsoil root length divided by subsoil root length	
Root growth rate	RGR	Average taproot growth rate	cm d^–1^
Root mass	RM	Root dry mass	mg
Shoot mass	SM	Shoot dry mass	mg
Root mass ratio	RMR	Root-to-shoot dry mass ratio	
Shoot height	SH	Shoot (main stem) height	cm
Leaf branch number	LBN	Number of leaf branches (off the main stem) per plant	

### Data analysis

General linear model (GLM) multivariate analysis was performed for genotype main effects after non-significant differences between bins and harvest times were identified using SPSS Statistics (Version 19, IBM, USA). General correlations between parameters were examined using Pearson correlation coefficients. Correlations were considered statistically significant at *P*≤0.05. Root traits with coefficient of variation (CV) values ≥0.3 were selected for principal component analysis (PCA) to identify determinants of root architecture variability across genotypes (Jolliffe, 2002). Hierarchical cluster analysis was used to determine variance among selected root traits and homogeneous groups among genotypes using the average linkage method. Mean data for selected root traits were subjected to *k*-means clustering analysis to generate relatively homogeneous groups of the tested genotypes. Further statistical analyses were performed on each group of genotypes separated by seed types (desi, kabuli, and pea-shaped) to show trait variation within each group and among the three seed-type groups. Additional descriptive data for each seed-type group are given in Supplementary Table S2.

## Results

### Rooting pattern and variation in root traits

**Fig. 1.**
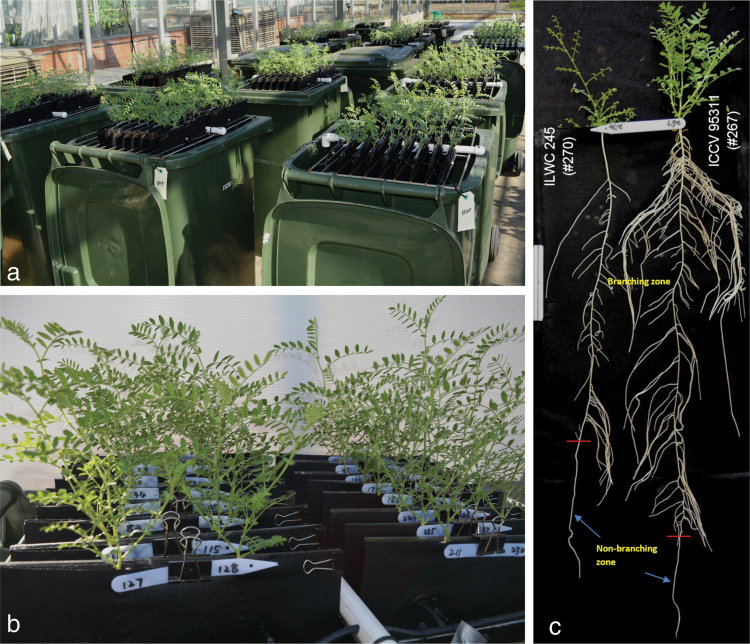
Experimental layout (a) and a close-up view (b) of chickpea plants grown in the semi-hydroponic phenotyping platform for characterising root trait variability in the germplasm in a temperature-controlled glasshouse, with an example (c) of two contrasting genotypes grown for 35 d: the white tag on the left-hand side is 10cm long. The branching zone (z1) and non-branching zone (z2) on the taproots are indicated.

The rooting pattern of the tested chickpea genotypes was generally dominated by the taproot (i.e. the main primary root) and the lateral roots (root branches), primarily first-order and second-order laterals, and third-order laterals in a few genotypes (Fig. 1). Genotypes exhibited large variation in rooting patterns and branching manner. Some genotypes had specific root traits such as long lateral roots, deeper roots with sparse and short branches, and fine roots (see Fig. 1 for an example root image). The two wild relatives, ILWC 235 and ILWC 245 (*C. echinospermum*) had similar root patterns to the desi-type of *C. arietinum*, but ILWC 245 was much smaller in root size with fewer lateral roots (ranked 164th in RL), and ILWC 235 was relatively shallow (ranked 173rd in TRL).

Root morphological and architectural traits differed significantly among genotypes. All of the measured root-related traits differed significantly among genotypes (*P*≤0.001 for 28 traits, *P*≤0.05 for TRL_z2) except for the root mass ratio (RMR; *P*= 0.275) (Table 2). Seventeen root traits had CVs >0.3, of which total branch number (BN), branch density (BD), root length in section 3 (RL_s3), subsoil root length (RL_sub), and subsoil branch length (BL_sub) had CVs >0.5.

### Root development and branching

Most chickpea genotypes developed roots vigorously. The average root growth rate (based on taproot elongation) was 2.06cm d^−1^ and ranged from 1.1 to 3.0cm d^−1^ (Table 2). Rooting depth ranged from 38.3cm (ICC 4567) to 105cm (ICC 16374) with a median taproot length of 72.2cm (Table 2; Supplementary Fig. S1). Total root length and root length in each 20-cm section varied significantly among genotypes (Table 2). Total root length (RL) ranged from 305cm (ICC 4567) to 3824cm (ICC 00316), with an average root length of 1175cm plant^−1^, and 37%, 36%, and 27% of the total root length being distributed in the 0−20, 20−40, and 40−110cm sections, respectively (Fig S2). The root length ratio (topsoil over subsoil) averaged 0.7 across all genotypes.

**Table 2.** Descriptive statistics of 33 measured traits (30 root traits, and three shoot traits) in 270 chickpea genotypes grown in a semi-hydroponic phenotyping platform

Trait	Minimum	Maximum	Mean	Median	Std. Deviation	CV*	*P* value*
TRL_z1	17.0	80.0	55.7	55.6	11.7	0.21	**<0.001**
TRL_z2	2.67	36.3	16.4	16.0	5.97	**0.37**	*0.042*
TRL	38.3	105	72.1	72.2	12.2	0.17	**0.001**
RL	305	3824	1175	1078	453	**0.42**	**<0.001**
BL	267	3765	1103	1004	447	**0.44**	**<0.001**
BN	23.1	764	188	159	105	**0.66**	**<0.001**
ABL	4.11	14.0	6.93	6.85	1.32	0.21	**<0.001**
RA	94.8	1027	319	294	123	**0.42**	**<0.001**
RV	2.36	22.03	6.95	6.38	2.75	**0.43**	**<0.001**
RD	0.68	1.07	0.87	0.87	0.06	0.07	**<0.001**
SRL	32.5	264	65.7	58.7	27.2	**0.46**	**<0.001**
BLR_tap	6.62	77.8	15.6	14.4	6.63	**0.46**	**<0.001**
BD	0.57	15.7	2.61	2.28	1.52	**0.67**	**<0.001**
BI	0.08	0.24	0.16	0.15	0.03	0.20	**<0.001**
RTD	1.27	9.87	3.05	3.05	0.82	0.27	**<0.001**
RL_top	140	923	440	426	122	0.29	**<0.001**
BL_top	120	903	420	406	122	**0.30**	**<0.001**
RD_top	0.77	1.09	0.92	0.92	0.06	0.06	**<0.001**
RL_s2	71.6	868	426	574	158	0.28	**<0.001**
RD_s2	0.64	1.00	0.82	0.82	0.06	0.08	**<0.001**
RL_s3	21.1	827	309	431	273	**0.63**	**<0.001**
RD_s3	0.62	1.05	0.86	0.86	0.08	0.09	**<0.001**
RL_sub	144	1575	735	647	378	**0.58**	**<0.001**
BL_sub	126	1497	683	597	372	**0.62**	**<0.001**
RD_sub	0.64	1.03	0.84	0.84	0.07	0.08	**<0.001**
RLR_top/sub	0.23	2.22	0.70	0.65	0.30	**0.46**	**<0.001**
BLR_top/sub	0.23	2.63	0.73	0.67	0.33	**0.49**	**<0.001**
RGR	1.10	3.00	2.06	2.06	0.35	0.17	**0.001**
RM	119	370	198	190	61.8	**0.32**	**<0.001**
SM	81.9	1006	329	297	142	**0.48**	**<0.001**
RMR	0.19	1.55	0.68	0.68	0.21	**0.31**	0.275
SH	8.23	30.0	16.8	17.0	4.26	0.25	**<0.001**
LBN	10.0	55.7	13.6	13.3	3.64	0.27	0.710

* Traits with coefficients of variation (CVs) ≥0.3 appear in bold type. Probability values (*P*) were based upon a GLM multivariate analysis of 270 genotypes and appear in bold if <0.01 and italic if <0.05 (see Table 1 for units of each trait).

Root branching differed among genotypes; TRL_z1 ranged from 17 to 80cm, accommodating 23 to 764 lateral roots (average 188), mostly first-order branches (Table 2). The average BD was 2.61cm^−1^ taproot length, ranging from 0.57 to 15.7, and BI ranged from 0.08 to 0.24 (mean 0.16) cm^−1^ root length.

### Root diameter and specific root length

**Fig. 2.**
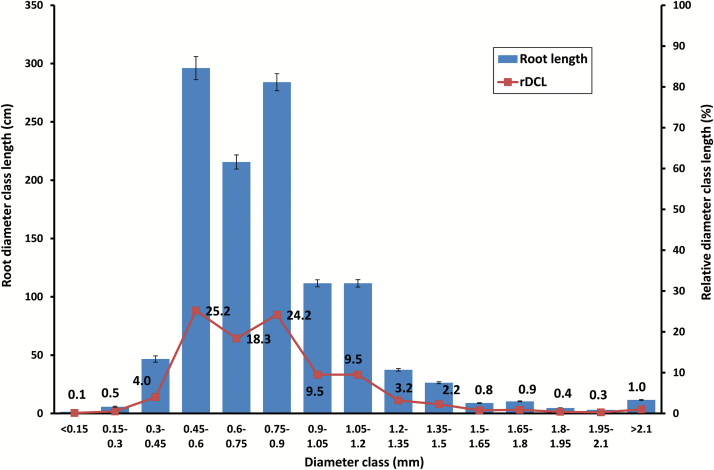
Root diameter class length (DCL, cm, mean ±SE) and relative diameter class length (rDCL,%) among 270 chickpea genotypes grown in a semi-hydroponic phenotyping platform. Percentage values for rDCL at each diameter class are given.

**Fig. 3.**
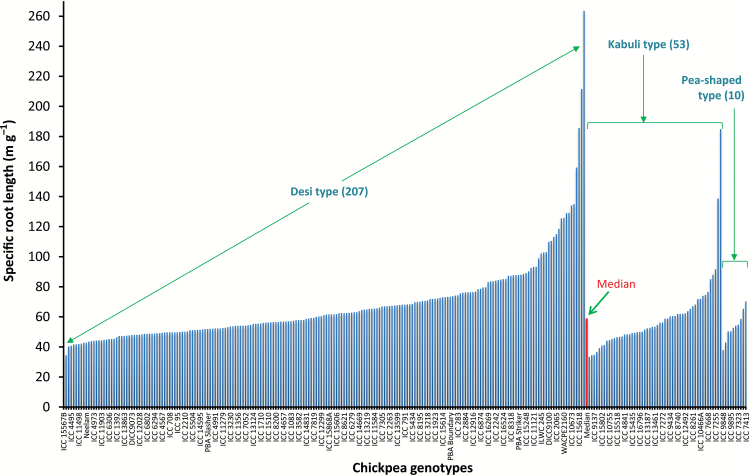
Genotypic variation in specific root length (SRL) among 270 chickpea genotypes (plotted by seed type: 207 desi, 53 kabuli, and 10 pea-shaped) grown in a semi-hydroponic phenotyping platform. The median value for all genotypes is also presented.

Chickpea genotypes had relatively thin roots with an average root diameter of 0.87mm. The variation in RD among genotypes was low (CV=0.07) (Table 2). Most of the root length was in the 0.45 to 0.9mm diameter classes, accounting for 67.7% of total root length (Fig. 2), with about 19% between 0.9 and 1.2mm. Roots thicker than 1.5mm, some 2.5% of the total roots, were primarily proximal (at the top, near the shoot). SRL varied from 32.5 to 264 m g^−1^ dry mass (mean 65.7) (Table 2; Fig. 3). RTD ranged from 1.3 to 9.9mg cm^−3^ (mean 3.1).

### Shoot parameters

Shoot-related traits, i.e. shoot height (SH), leaf branch numbers (off the main stem, LBN) and shoot dry mass (SM), were measured at harvest (35 d after sowing). ANOVA identified significant differences among genotypes in SH and SM (*P*<0.001), but not in LBN (*P=*0.71) (Table 2). SM had a CV value >0.3 but the other two shoot traits had low CV values.

### Correlation among traits

**Fig. 4.**
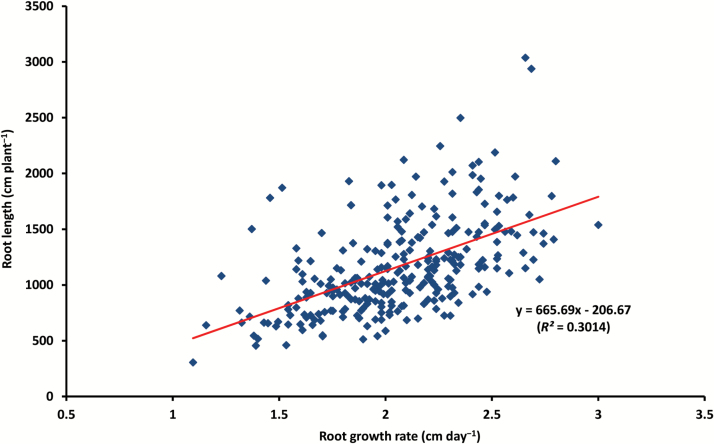
Correlation between root growth rate and root length in 270 chickpea genotypes. Mean values of three replicates are plotted.

**Fig. 5.**
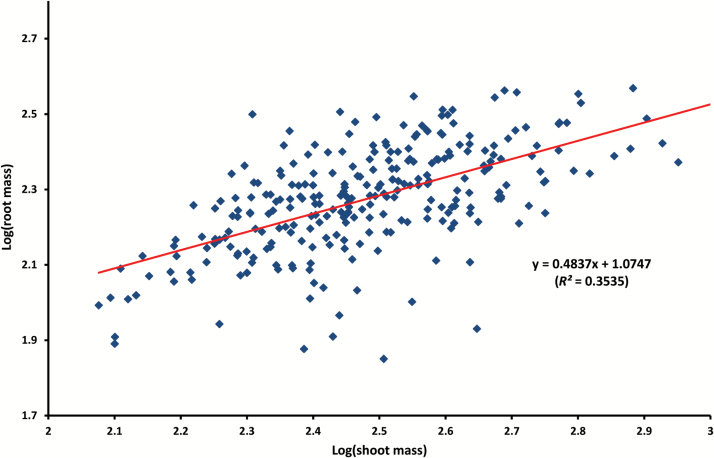
Correlation between root dry mass and shoot dry mass among 270 chickpea genotypes grown in the semi-hydroponic phenotyping platform. Data are logarithmic means of three replicates.

**Fig. 6.**
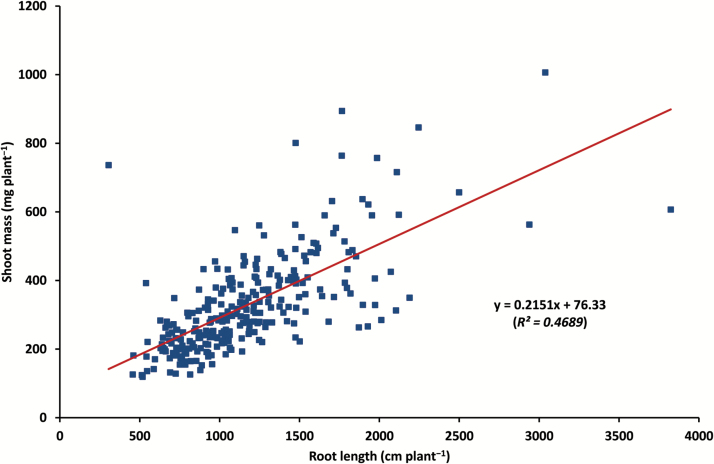
Correlation between root length and shoot dry mass among 270 chickpea genotypes grown in the semi-hydroponic phenotyping platform. Mean values of three replicates are plotted.

A subset of 17 root traits and one shoot trait (shoot mass) with relatively large coefficients of variation (≥0.3, Table 2) was selected for Pearson correlation analysis to identify relationships among the measured traits. The Pearson correlation matrix showed strong correlations among most of the selected traits (Table 3). For example, total root length (RL), total branch length (BL), and branch number (BN) were strongly associated with all other traits (all *P*<0.001). Specific root length (SRL) was positively correlated with RL, BL, BN, root area and volume, and negatively associated with RLR_top/sub, BLR_top/sub, and RM (*P*<0.001). In contrast, taproot length at the non-branching zone (TRL_z2) was strongly correlated with SRL, RM, and RMR (all *P*<0.001), BLR_tap, BD, and RLR_top/sub (all *P*<0.05). RL was positively correlated with RM (*P*<0.01; Table 3). RL showed a strong correlation with RGR (*P*<0.01, Fig. 4), which reflects the relationship between RL and root depth at harvest since RGR was based on the taproot growth. There was a general trend of greater root length with the increased root growth rate; however, genotypes with deeper roots did not always have larger root systems in terms of total root length, and vice versa. A strong correlation was found between SM and each of the selected 17 root traits except for TRL-z2, SRL, RLR_top/sub, and BLR_top/sub (*P*<0.001, Table 3). The correlation between RM and SM was also significant (*R*^2^=0.35, *P*<0.01, Fig. 5). Root length was positively associated with shoot mass (*R*^2^=0.47, *P*<0.01, Fig. 6).

**Table 3.** Pearson’s correlation matrix for 17 root traits and one shoot trait (shoot mass, SM) in 270 chickpea genotypes.

	TRL_z2	RL	BL	BN	RA	RV	SRL	BLR_tap	BD	BL_top	RL_s3	RL_sub	BL_sub	RLR_top/sub	BLR_top/sub	RM	SM	RMR
TRL_z2		**0.00**	–0.01	–0.04	0.04	0.08	–0.24	–0.16	–0.15	–0.04	–0.02	0.01	0.00	–0.12	–0.11	0.23	–0.08	0.33
RL	0.978		1.00	0.97	0.98	0.94	0.48	0.84	0.87	0.69	0.87	0.97	0.97	–0.54	–0.54	0.55	0.48	–0.29
BL	0.861	**0.000**		0.97	0.98	0.94	0.49	0.85	0.88	0.70	0.87	0.97	0.97	–0.54	–0.54	0.54	0.48	–0.29
BN	0.521	**0.000**	**0.000**		0.93	0.86	0.54	0.81	0.90	0.69	0.80	0.93	0.93	–0.50	–0.50	0.48	0.48	–0.34
RA	0.521	**0.000**	**0.000**	**0.000**		0.99	0.40	0.84	0.84	0.69	0.85	0.95	0.95	–0.52	–0.51	0.60	0.50	–0.26
RV	0.210	**0.000**	**0.000**	**0.000**	**0.000**		0.32	0.81	0.79	0.67	0.82	0.91	0.91	–0.47	–0.47	0.64	0.51	–0.23
SRL	**0.000**	**0.000**	**0.000**	**0.000**	**0.000**	**0.000**		0.53	0.59	0.20	0.55	0.51	0.52	–0.37	–0.36	–0.30	0.11	–0.48
BLR_tap	*0.011*	**0.000**	**0.000**	**0.000**	**0.000**	**0.000**	**0.000**		0.96	0.59	0.76	0.82	0.83	–0.36	–0.36	0.37	0.37	–0.28
BD	*0.014*	**0.000**	**0.000**	**0.000**	**0.000**	**0.000**	**0.000**	**0.000**		0.62	0.75	0.84	0.85	–0.38	–0.39	0.36	0.40	–0.33
BL_top	0.497	**0.000**	**0.000**	**0.000**	**0.000**	**0.000**	**0.001**	**0.000**	**0.000**		0.33	0.51	0.51	0.04	0.03	0.61	0.54	–0.32
RL_s3	0.720	**0.000**	**0.000**	**0.000**	**0.000**	**0.000**	**0.000**	**0.000**	**0.000**	**0.000**		0.93	0.93	–0.61	–0.60	0.29	0.28	–0.19
RL_sub	0.852	**0.000**	**0.000**	**0.000**	**0.000**	**0.000**	**0.000**	**0.000**	**0.000**	**0.000**	**0.000**		1.00	–0.66	–0.66	0.46	0.41	–0.24
BL_sub	0.990	**0.000**	**0.000**	**0.000**	**0.000**	**0.000**	**0.000**	**0.000**	**0.000**	**0.000**	**0.000**	**0.000**		–0.66	–0.65	0.45	0.40	–0.24
RLR_top/ sub	*0.048*	**0.000**	**0.000**	**0.000**	**0.000**	**0.000**	**0.000**	**0.000**	**0.000**	0.545	**0.000**	**0.000**	**0.000**		1.00	–0.20	–0.07	–0.04
BLR_top/ sub	0.075	**0.000**	**0.000**	**0.000**	**0.000**	**0.000**	**0.000**	**0.000**	**0.000**	0.646	**0.000**	**0.000**	**0.000**	**0.000**		–0.20	–0.07	–0.04
RM	**0.000**	**0.000**	**0.000**	**0.000**	**0.000**	**0.000**	**0.000**	**0.000**	**0.000**	**0.000**	**0.000**	**0.000**	**0.000**	**0.001**	**0.001**		0.39	0.25
SM	0.187	**0.000**	**0.000**	**0.000**	**0.000**	**0.000**	0.067	**0.000**	**0.000**	**0.000**	**0.000**	**0.000**	**0.000**	0.271	0.268	**0.000**		–0.53
RMR	**0.000**	**0.000**	**0.000**	**0.000**	**0.000**	**0.000**	**0.000**	**0.000**	**0.000**	**0.000**	**0.002**	**0.000**	**0.000**	0.534	0.530	**0.000**	0.000	

Traits with CVs ≥0.3 were included in the analysis (see Table 2). Proportion values are given on the lower left side of the matrix table (in bold if <0.01 and italic if <0.05, indicating significance of correlation).

### Determination of root trait variability

Seventeen root traits with CVs ≥0.3 (Table 2) were included in the principal component analysis. Three principal components (PCs) were identified with eigenvalues >1, capturing 81.5% of the total variation in root system architectural traits across the 270 chickpea genotypes (Table 4). The first component (PC1) represented 56% of the variability and accounted primarily for global root traits such as BL, RL, BN, RA, RV, BD, and root length and branch length in the topsoil and subsoil. PC2 represented 14.7% of the variation derived from ratios of branch length and root length in the topsoil and subsoil (BLR_top/sub and RLR_top/sub). The third component (PC3, 10.8% variation) mainly accounted for SRL, TRL_z2, RM, and RMR.

**Table 4.** Variable loading scores of 17 root traits and the proportion of variation of each principal component.

	PC1	PC2	PC3
BL	**0.98**	0.04	–0.03
RL	**0.98**	0.03	–0.02
BN	**0.97**	0.00	–0.10
RA	**0.97**	0.06	0.06
RV	**0.93**	0.09	0.13
BD	**0.90**	0.12	–0.26
BL_sub	**0.89**	–0.32	0.08
BLR_tap	**0.85**	0.20	–0.23
BL_top	**0.74**	0.49	0.24
RL_top	**0.74**	0.49	0.24
RL_s3	**0.71**	–0.46	0.03
BLR_top/sub	–0.40	**0.87**	0.01
RLR_top/sub	–0.40	**0.88**	0.01
SRL	0.47	0.00	**–0.75**
RM	0.59	0.08	**0.71**
TRL_z2	–0.02	–0.22	**0.58**
RMR	–0.25	–0.21	**0.40**
Eigenvalue	9.52	2.50	1.83
Variability (%)	56.0	14.7	10.8
Cumulative variability (%)	56.0	70.7	81.5

Seventeen root traits with CVs ≥0.3 (see Table 1) were used for factor analysis using the principal component analysis (PCA) extraction method. Rotation converged in 17 iterations using Varimax with Kaiser Normalization. For each trait, the largest variable loading score crossing the three components appears in bold. Principal components with eigenvalues >1 are presented and considered significant (Tabachnik and Fidell, 1996).

**Fig. 7.**
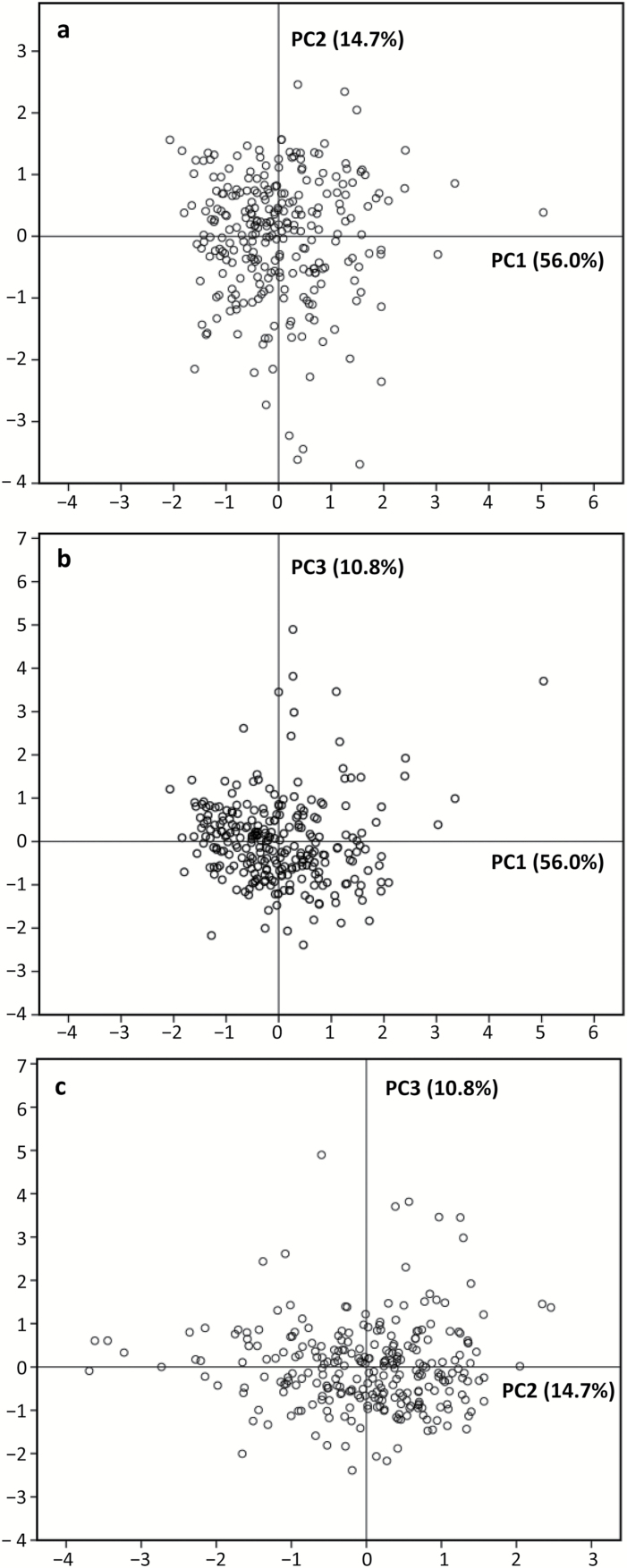
Principal component analysis of 17 selected root traits with CVs ≥0.3 among 270 chickpea genotypes grown in a semi-hydroponic phenotyping platform. The position of each genotype is shown for principal component PC1 vs. PC2 representing 70.7% of the variability (a), PC1 vs. PC3 representing 66.8% of the variability (b), and PC2 vs. PC3 representing 25.5% of the variability (c).

Genotype distribution based on PCA regression scores of the 17 selected root traits is shown in Fig. 7. The relative distance among the 270 genotypes is displayed for each combination of root traits. Loading plots for PC1 vs. PC2, PC1 vs. PC3, and PC2 vs. PC3 represented 70.7%, 66.8%, and 25.5% of the variability, respectively. The two plots with PC1 (Fig. 7a, b) show that one genotype appears as an outlier with the largest RL, BL and BN, RL_top, BL_top and BN_top. This genotype is ICC 00316B according to the root trait loading score of PC1 (Table 4) and the root data, such as RL (Supplementary Fig. S2). The plot of PC2 vs. PC3 separates genotypes into RLR_top/sub and BLR_top/sub (PC2) and SRL, RM TRL_Z2 and RMR (PC3, Fig. 7c).

**Fig. 8.**
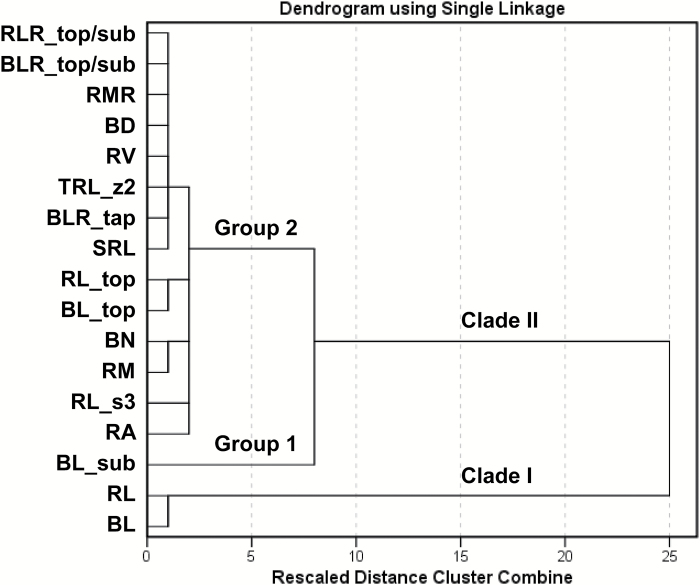
Dendrogram showing clustering patterns of 17 selected root traits with CVs ≥0.3 among 270 chickpea genotypes grown in a semi-hydroponic phenotyping platform. Hierarchical cluster analysis was carried out using the average linkage (between groups) method (see Table 1 for trait descriptions, and Table 2 for CV values).

Hierarchical cluster analysis on the same set of root traits using the average linkage method clearly distinguished two general clades: Clade I contains RL and BL, while Clade II contains the remaining 15 traits (Fig. 8). The dendrogram indicates that Clade II comprises two major groups by separating BL_sub from the other traits.

### Genotype homogeneous grouping based on root trait variation

**Fig. 9.**
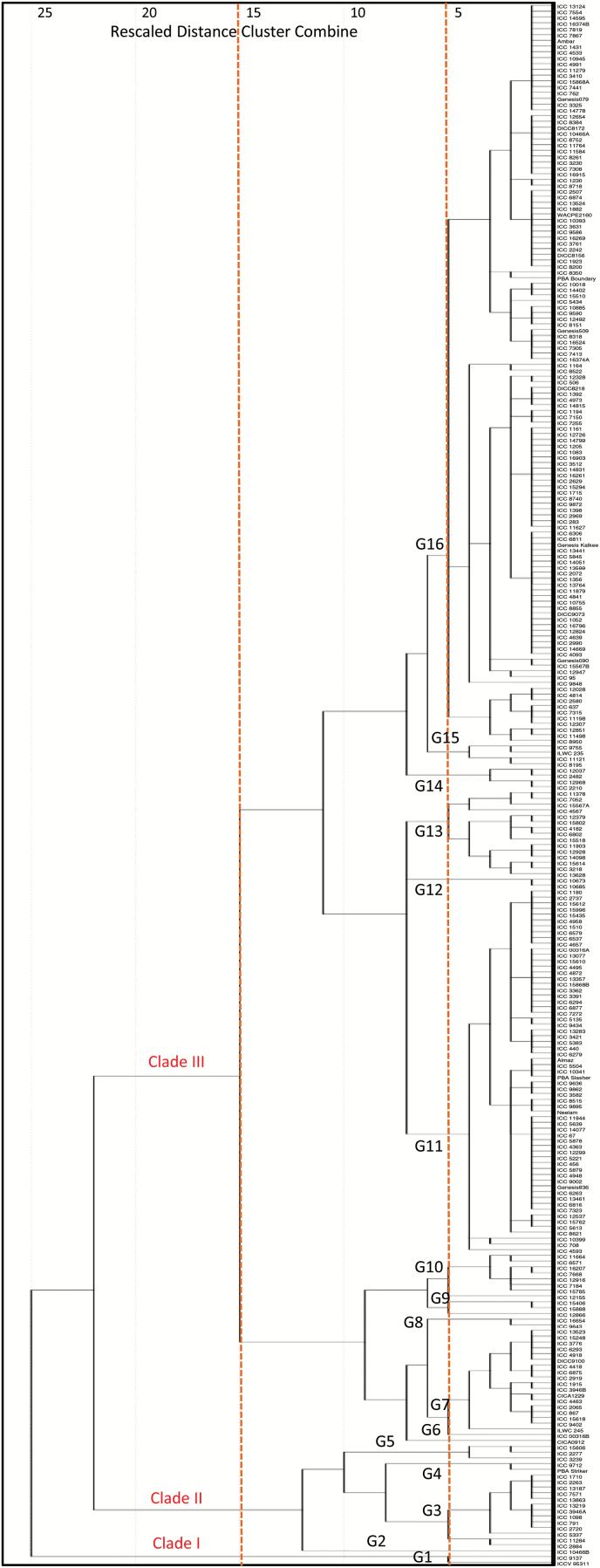
Dendrogram of agglomerative hierarchical clustering (AHC) using the Pearson correlation coefficient on 17 selected root traits with CVs ≥0.3. The 270 chickpea genotypes were assigned to one of three general clades (Clade I, II, or III) at a rescaled distance of 15 (left dashed line) containing 16 groups (G1 to G16) at a rescaled distance of 5 (right dashed line). The 17 root traits are the same as those used for PCA in Table 4.

An agglomerative hierarchical clustering (AHC) dendrogram was constructed using the Pearson correlation coefficients of the 17 selected root traits and exhibits large diversity in traits among the 270 chickpea genotypes (Fig. 9). Three general clades (Clade I, II, and III) were determined using a rescaled distance of 15, which can be further separated into 16 sub-groups (G1 to G16) at a rescaled distance of 5. Clade I contains two genotypes, ICC 9137 (landrace from Iran) and ICCV 95311 (breeding material from India). Clade II embraces 16 genotypes from four sub-groups. The largest Clade (III) contains 252 genotypes from 11 sub-groups (G6−G16) and the number of genotypes in each sub-group varies from 1 (G6) to 126 (G16).

### Genotypic variation in root traits for seed types

The experimental data were further analysed based on seed types (207 desi, 53 kabuli, and 10 pea-shaped). There were small differences in trait variation among these types determined by the CV values, as shown in the descriptive statistics presented in Supplementary Table S2. For example, 17 root traits in desi, 20 in kabuli, and 15 in pea-shaped seeds had relatively high variations with CV ≥0.3. The largest variation in RL was found in desi and ranged from 305 to 3824, followed by kabuli (from 539 to 2938), and pea-shaped (546 to 1271) (Supplementary Table S2 and Fig. S1). The root systems of the pea-shaped genotypes were shallower with less variation in TRL, and the desi and kabuli types had similar root depth, except one deep-rooting desi genotype (ICC 16374B) (Fig. S2). Two desi types (ICC 12155 and ILWC 235) had the largest SRL values amongst the genotypes, whilst the 10 pea-shaped genotypes had relatively thicker roots, as indicated by smaller SRL values (Fig. 3).

## Discussion

The search for root traits conferring efficiency in resource acquisition and adaptation to edaphic stresses, particularly in drying soil environments, has increased in both scientific research and breeding programs. Progress has been hampered due to the difficulties in phenotyping root traits efficiently and accurately, and the wide-scale use of root genetic information in breeding programs remains a challenge (de Dorlodot *et al.*, 2007; Ghanem *et al.*, 2011; Chen *et al.*, 2015). Using a recently developed novel semi-hydroponic phenotyping system (Chen *et al.*, 2011*a*), this study established the existence of large variations in various root traits in a core collection of 270 genotypes of chickpea (Table 2, Fig. 1, Supplementary Table S2 and Figs S1, S2). Trait data collected using this system exhibited no serious departures from multivariate normality (the multivariate standard errors of skewness and kurtosis were 0.148 and 0.295, respectively, when all parameters were included in the GLM analysis). The phenotyping platform provided quality data for assessing intrinsic genetic variations in root traits among the tested chickpea genotypes, as shown in our recent root studies in narrow-leafed lupin (Chen *et al.*, 2011*a*, *b*, 2012, 2016), wheat, and barley (Yinglong Chen, unpublished data) using the same phenotyping platform. Root trait data acquired in the present study form the basis for paramaterising three-dimensional root structural–functional models, such as ROOTMAP (Diggle, 1988; Dunbabin *et al.*, 2013), and SimRoot (Lynch *et al.*, 1997), for rapid and reliable reconstruction of root systems, similar to our recent simulation studies in narrow-leafed lupin (Chen *et al.*, 2011*b*, 2013*b*).

The root system of chickpea is characterised by a taproot-dominant rooting pattern comprising first-order lateral roots and the presence of densely or sparsely distributed second-order branches (Fig. 1), similar to the root system of narrow-leafed lupin (Chen *et al.*, 2012). However, the 270 genotypes tested displayed large diversity in their rooting patterns. For example, the deep-rooting genotypes had nearly twice the taproot length as the shallow-rooting genotypes (Table 2, Supplementary Fig. S1). The genotypes with larger root systems had root branching zones (TRL_z1) that were approximately four times longer and BD values that were 26 times higher than the smallest genotype (Table 2). Rooting depth and root branch density are important root architectural traits that directly influence the acquisition of water and nutrients in the soil strata (e.g. Lynch and Wojciechowski, 2015). Studies show that a prolific root system in chickpea is closely associated with grain yield under terminal drought conditions (Kashiwagi *et al.*, 2006; Varshney *et al.*, 2013).

Significant genetic variation in root length density and root dry weight has been previously observed in 257 recombinant inbred lines (RILs) derived from a cross between a breeding line ICC 4958 (large root system) and ‘Annigeri’ (an agronomically preferred variety) evaluated 35 d after sowing in a field trial under terminal drought (Serraj *et al.*, 2004). In a previous study using a cylinder culture system that tested 216 chickpea genotypes, ICC 8261 and ICC 4958 exhibited prolific and deep roots, while ICC 1882 and ICC 283 had less prolific and shallower roots (Kashiwagi *et al.*, 2005). Our study showed that ICC 8261 and ICC 4958 developed deeper roots (ranked 35th and 54th, respectively, in taproot length of 270 genotypes), while ICC 283 had a median root depth (ranked 149th) and ICC 1882 had relatively deeper roots (ranked 26th) (Table 2, Supplementary Fig. S1). Some of the observed differences in taproot length between the two studies may be explained by the different growth media used.

As an important food legume crop, chickpea is mostly grown on residual moisture from monsoon rains on the Indian subcontinent and semiarid regions of Sub-Saharan Africa (Varshney *et al.*, 2013; Foyer *et al.*, 2016). Drought stress, particularly at the end of the growing season, is a major constraint limiting chickpea production and yield stability in arid and semiarid regions of the world (Krishnamurthy *et al.*, 2010; Varshney *et al.*, 2013; Kashiwagi *et al.*, 2015; Pang *et al.* 2016). The chickpea genotypes differing in root proliferation and branching identified in this study may perform differently in terms of plant foraging and water and nutrient capture, particularly when resources are sparsely and heterogeneously distributed at different soil depths. This study identified deep-rooting genotypes, such as ICC 16374B, ICC 15510, ICC 9586, and ICC 867 (Supplementary Fig. S1), which may have the advantage of accessing subsoil reserves of water when the topsoil dries out later in the season in Mediterranean regions, including large parts of the southern and western Australian cereal belt (Siddique and Sedgley, 1986; Siddique *et al.*, 2000; Carvalho *et al.*, 2014; Lynch and Wojciechowski, 2015). Therefore, the capacity of roots to grow into the subsoil is significant for avoiding terminal drought stress in water-limited soils (Serraj *et al.*, 2004). In addition to root architectural traits, some root anatomical traits such as root cortex and root cortical aerenchyma are linked to drought resistance (Jaramillo *et al.*, 2013; Chimungu *et al.*, 2015). In this study, chickpea plants grown in a semi-hydroponic system supplied with a relatively low-level of nutrients may perform differently under soil conditions. Our recent studies in narrow-leafed lupin showed consistent ranking for genotypes for a number of important root traits grown in various environments, including three different soil types under controlled and field conditions in Western Australia, suggesting the reliability of the semi-hydroponic phenotyping platform in providing basic data for characterising root trait variability in germplasm under no stressed conditions (Chen *et al.*, 2011*b*, 2012, 2014, 2016). Genotypes with contrasting root traits identified in this study can be selected to assess their adaption to drought stress, low soil fertility, and other edaphic stresses in follow-up studies, as undertaken in narrow-leafed lupin (Chen *et al.*, 2011*a*, *b*, 2012, 2013*a*, *b*, 2014, 2016).

The complexity of crop root systems requires a better understanding of the multiple associations among root traits (Lynch, 1995; Zhu *et al.*, 2011). This study explored Pearson’s correlation analysis, principal component analysis, and hierarchical cluster analysis, and clearly demonstrated (1) the relationship among some root traits (Tables 3, 4), (2) the relative contribution of individual root traits (Fig. 7), and (3) the determinants for genotype grouping based on relatively homogeneous root traits (Fig. 8). The results highlighted genotype groups that could be crossed to identify the genetic basis of specific root traits, which may help to characterise those traits suitable for targeted genotype selection and breeding of new chickpea varieties for efficient use of water and nutrients.

Phenotyping for root trait properties in extensive germplasm is essential for breeding new varieties through marker-assisted selection (MAS) programs. Root trait-marker association and linkage mapping analyses have already identified the genetic basis of a few root traits in chickpea (Varshney *et al.*, 2013; Mahendar *et al.*, 2014; Thudi *et al.*, 2014*a*, *b*, 2016; Varshney, 2016), and some traits in rice (Courtois *et al.*, 2013; Henry, 2013), maize (Manavalan *et al.*, 2011), durum wheat (Sanguineti *et al.*, 2007), barley (Naz *et al.*, 2012; Arifuzzaman *et al.*, 2014), and narrow-leafed lupin (Chen *et al.*, 2016). In chickpea, analyses of quantitative trait loci (QTLs) have identified a so-called ‘*QTL-hotspot*’ genomic region on linkage group 4 (CaLG04) harbouring QTLs for several root and drought-tolerance traits (Varshney *et al.*, 2014). Previously, a QTL for deep rooting, DEEPER ROOTING 1 (DRO1) controlling steep root growth angles, was mapped to chromosome 9 in the rice progeny of a shallow-rooting IR64 and deep-rooting KP cross (Uga *et al.*, 2011).

In conclusion, the phenotyping study described here identified wide variation in root system architectural traits across a large sample of chickpea germplasm. For the first time, chickpea genotypes with vastly different root properties were characterised for further studies ultimately aimed at developing breeding lines with root traits for improved adaptation to specific environments. The present study and follow-up investigations in field or glasshouse trials using molecular markers and QTL mapping are expected to identify candidate genotypes with suitable root traits for potential breeding for efficient water and nutrient capture in stressful or poor soil environments. In addition to root trait phenotyping and genotyping, root structural–functional models are a promising tool for selecting superior genotypes with optimised root systems for adaptation to target environments.

## Supplementary data

Supplementary data are available at *JXB* online.

Table S1. Country of origin and seed type of the 270 chickpea genotypes used in this study.

Table S2. Descriptive statistics for seed types (desi, kabuli, and pea-shaped) of 33 measured traits (30 root traits and three shoot traits) in 270 chickpea genotypes.

Figure S1. Genotypic variation in taproot length among 270 chickpea genotypes plotted by seed types (desi, kabuli, and pea-shaped).

Figure S2. Genotypic variation in total root length among 270 chickpea genotypes plotted by seed types (desi, kabuli, and pea-shaped).

## Supplementary Material

Supplementary_Tables_S1_S2_figures_S1_S2
