# Supplementary material for: Characterising root trait variability in chickpea (Cicer arietinum L.) germplasm
Source: J Exp Bot. 2016 Oct 6;68(8):1987–99. doi: 10.1093/jxb/erw368 (PMC5429021; doi:10.1093/jxb/erw368)
Supplement: Supplementary_Tables_S1_S2_figures_S1_S2 [file erw368_suppl_Supplementary_Tables_S1_S2_figures_S1_S2.pdf]

Supplementary data

Table S1. Country of origin and seed type of 270 chickpea genotypes used in this study

Genotype	Origin	Seed type	Species
**Almaz**	Australia	Kabuli	*Cicer arietinum*
**Ambar**	Australia	Desi	*Cicer arietinum*
**CICA0912**	Australia	Desi	*Cicer arietinum*
**CICA1229**	Australia	Desi	*Cicer arietinum*
**DICC8156**	Australia	Desi	*Cicer arietinum*
**DICC8172**	Australia	Desi	*Cicer arietinum*
**DICC8218**	Australia	Desi	*Cicer arietinum*
**DICC9073**	Australia	Desi	*Cicer arietinum*
**DICC9100**	Australia	Desi	*Cicer arietinum*
**Genesis Kalkee**	Australia	Kabuli	*Cicer arietinum*
**Genesis079**	Australia	Kabuli	*Cicer arietinum*
**Genesis090**	Australia	Kabuli	*Cicer arietinum*
**Genesis509**	Australia	Desi	*Cicer arietinum*
**Genesis836**	Australia	Desi	*Cicer arietinum*
**ICC 00316A**	Australia	Desi	*Cicer arietinum*
**ICC 00316B**	Australia	Desi	*Cicer arietinum*
**ICC 10018**	India	Desi	*Cicer arietinum*
**ICC 10341**	Turkey	Pea-shaped	*Cicer arietinum*
**ICC 10393**	India	Desi	*Cicer arietinum*
**ICC 10399**	India	Desi	*Cicer arietinum*
**ICC 10466A**	India	Kabuli	*Cicer arietinum*
**ICC 10466B**	India	Kabuli	*Cicer arietinum*
**ICC 1052**	Pakistan	Desi	*Cicer arietinum*
**ICC 10673**	Turkey	Desi	*Cicer arietinum*
**ICC 10685**	Turkey	Desi	*Cicer arietinum*
**ICC 10755**	Turkey	Kabuli	*Cicer arietinum*
**ICC 1083**	Iran	Desi	*Cicer arietinum*
**ICC 10885**	Ethiopia	Kabuli	*Cicer arietinum*
**ICC 10945**	India	Desi	*Cicer arietinum*
**ICC 1098**	Iran	Desi	*Cicer arietinum*
**ICC 11121**	India	Desi	*Cicer arietinum*
**ICC 11198**	India	Desi	*Cicer arietinum*
**ICC 11279**	Pakistan	Desi	*Cicer arietinum*
**ICC 11284**	USSR	Desi	*Cicer arietinum*
**ICC 11378**	India	Desi	*Cicer arietinum*
**ICC 11498**	India	Desi	*Cicer arietinum*
**ICC 11584**	India	Desi	*Cicer arietinum*
**ICC 1161**	Pakistan	Desi	*Cicer arietinum*
**ICC 11627**	India	Desi	*Cicer arietinum*
**ICC 1164**	Nigeria	Desi	*Cicer arietinum*
**ICC 11664**	India	Desi	*Cicer arietinum*
**ICC 11764**	Chile	Kabuli	*Cicer arietinum*
**ICC 1180**	India	Desi	*Cicer arietinum*
**ICC 11879**	Turkey	Kabuli	*Cicer arietinum*
**ICC 11903**	Germany	Desi	*Cicer arietinum*
**ICC 1194**	India	Desi	*Cicer arietinum*
**ICC 11944**	Nepal	Desi	*Cicer arietinum*
**ICC 12028**	Mexico	Desi	*Cicer arietinum*
**ICC 12037**	Mexico	Kabuli	*Cicer arietinum*
**ICC 1205**	India	Desi	*Cicer arietinum*
**ICC 12155**	Bangladesh	Desi	*Cicer arietinum*
**ICC 12299**	Nepal	Desi	*Cicer arietinum*
**ICC 1230**	India	Desi	*Cicer arietinum*
**ICC 12307**	Myanmar	Desi	*Cicer arietinum*
**ICC 12328**	Cyprus	Kabuli	*Cicer arietinum*
**ICC 12379**	Iran	Desi	*Cicer arietinum*
**ICC 12492**	India	Kabuli	*Cicer arietinum*
**ICC 12537**	Ethiopia	Desi	*Cicer arietinum*
**ICC 12654**	Ethiopia	Desi	*Cicer arietinum*
**ICC 12726**	Ethiopia	Desi	*Cicer arietinum*
**ICC 12824**	Ethiopia	Desi	*Cicer arietinum*
**ICC 12851**	Ethiopia	Desi	*Cicer arietinum*

**ICC 12866**	Ethiopia	Desi	*Cicer arietinum*
**ICC 12916**	India	Desi	*Cicer arietinum*
**ICC 12928**	India	Desi	*Cicer arietinum*
**ICC 12947**	India	Desi	*Cicer arietinum*
**ICC 12968**	India	Kabuli	*Cicer arietinum*
**ICC 13077**	India	Kabuli	*Cicer arietinum*
**ICC 13124**	India	Desi	*Cicer arietinum*
**ICC 13187**	Iran	Kabuli	*Cicer arietinum*
**ICC 13219**	Iran	Desi	*Cicer arietinum*
**ICC 13283**	Iran	Kabuli	*Cicer arietinum*
**ICC 13357**	Iran	Kabuli	*Cicer arietinum*
**ICC 13441**	Iran	Kabuli	*Cicer arietinum*
**ICC 13461**	Iran	Kabuli	*Cicer arietinum*
**ICC 13523**	Iran	Kabuli	*Cicer arietinum*
**ICC 13524**	Iran	Desi	*Cicer arietinum*
**ICC 1356**	India	Desi	*Cicer arietinum*
**ICC 13599**	Iran	Desi	*Cicer arietinum*
**ICC 13628**	Iran	Kabuli	*Cicer arietinum*
**ICC 13764**	Iran	Kabuli	*Cicer arietinum*
**ICC 13863**	Ethiopia	Desi	*Cicer arietinum*
**ICC 1392**	Ethiopia	Desi	*Cicer arietinum*
**ICC 1398**	India	Desi	*Cicer arietinum*
**ICC 14051**	Ethiopia	Desi	*Cicer arietinum*
**ICC 14077**	Ethiopia	Desi	*Cicer arietinum*
**ICC 14098**	Ethiopia	Desi	*Cicer arietinum*
**ICC 1431**	India	Desi	*Cicer arietinum*
**ICC 14402**	India	Desi	*Cicer arietinum*
**ICC 14595**	India	Desi	*Cicer arietinum*
**ICC 14669**	India	Desi	*Cicer arietinum*
**ICC 14778**	India	Desi	*Cicer arietinum*
**ICC 14799**	India	Desi	*Cicer arietinum*
**ICC 14815**	India	Desi	*Cicer arietinum*
**ICC 14831**	India	Desi	*Cicer arietinum*
**ICC 1510**	India	Desi	*Cicer arietinum*
**ICC 15248**	Iran	Desi	*Cicer arietinum*
**ICC 15294**	Iran	Kabuli	*Cicer arietinum*
**ICC 15406**	Morocco	Kabuli	*Cicer arietinum*
**ICC 15435**	Morocco	Kabuli	*Cicer arietinum*
**ICC 15510**	Morocco	Desi	*Cicer arietinum*
**ICC 15518**	Morocco	Kabuli	*Cicer arietinum*
**ICC 15567A**	India	Desi	*Cicer arietinum*
**ICC 15567B**	India	Desi	*Cicer arietinum*
**ICC 15606**	India	Desi	*Cicer arietinum*
**ICC 15610**	India	Desi	*Cicer arietinum*
**ICC 15612**	Tanzania	Desi	*Cicer arietinum*
**ICC 15614**	Tanzania	Desi	*Cicer arietinum*
**ICC 15618**	India	Desi	*Cicer arietinum*
**ICC 15762**	Syria	Desi	*Cicer arietinum*
**ICC 15785**	Syria	Desi	*Cicer arietinum*
**ICC 15802**	Syria	Kabuli	*Cicer arietinum*
**ICC 15868A**	India	Desi	*Cicer arietinum*
**ICC 15868B**	India	Desi	*Cicer arietinum*
**ICC 15888**	India	Pea-shaped	*Cicer arietinum*
**ICC 15996**	Ethiopian	Desi	*Cicer arietinum*
**ICC 16207**	Myanmar	Desi	*Cicer arietinum*
**ICC 16261**	Malawi	Desi	*Cicer arietinum*
**ICC 16269**	Malawi	Desi	*Cicer arietinum*
**ICC 16374A**	Malawi	Desi	*Cicer arietinum*
**ICC 16374B**	Malawi	Desi	*Cicer arietinum*
**ICC 16524**	Pakistan	Desi	*Cicer arietinum*
**ICC 16654**	China	Kabuli	*Cicer arietinum*
**ICC 16796**	Portugal	Kabuli	*Cicer arietinum*
**ICC 16903**	India	Desi	*Cicer arietinum*
**ICC 16915**	India	Desi	*Cicer arietinum*
**ICC 1710**	India	Desi	*Cicer arietinum*
**ICC 1715**	India	Desi	*Cicer arietinum*
**ICC 1882**	India	Desi	*Cicer arietinum*
**ICC 1915**	India	Desi	*Cicer arietinum*

**ICC 1923**	India	Desi	*Cicer arietinum*
**ICC 2065**	India	Desi	*Cicer arietinum*
**ICC 2072**	India	Desi	*Cicer arietinum*
**ICC 2210**	Algeria	Desi	*Cicer arietinum*
**ICC 2242**	India	Desi	*Cicer arietinum*
**ICC 2263**	Iran	Desi	*Cicer arietinum*
**ICC 2277**	Iran	Kabuli	*Cicer arietinum*
**ICC 2482**	Iran	Kabuli	*Cicer arietinum*
**ICC 2507**	Iran	Desi	*Cicer arietinum*
**ICC 2580**	Iran	Desi	*Cicer arietinum*
**ICC 2629**	Iran	Desi	*Cicer arietinum*
**ICC 2720**	Iran	Desi	*Cicer arietinum*
**ICC 2737**	Iran	Desi	*Cicer arietinum*
**ICC 283**	India	Desi	*Cicer arietinum*
**ICC 2884**	Iran	Desi	*Cicer arietinum*
**ICC 2919**	Iran	Desi	*Cicer arietinum*
**ICC 2969**	Iran	Desi	*Cicer arietinum*
**ICC 2990**	Iran	Desi	*Cicer arietinum*
**ICC 3218**	Iran	Desi	*Cicer arietinum*
**ICC 3230**	Iran	Desi	*Cicer arietinum*
**ICC 3239**	Iran	Desi	*Cicer arietinum*
**ICC 3325**	Cyprus	Desi	*Cicer arietinum*
**ICC 3362**	Iran	Desi	*Cicer arietinum*
**ICC 3391**	Iran	Desi	*Cicer arietinum*
**ICC 3410**	Iran	Kabuli	*Cicer arietinum*
**ICC 3421**	Israel	Kabuli	*Cicer arietinum*
**ICC 3512**	Iran	Desi	*Cicer arietinum*
**ICC 3582**	Iran	Desi	*Cicer arietinum*
**ICC 3631**	Iran	Desi	*Cicer arietinum*
**ICC 3761**	Iran	Desi	*Cicer arietinum*
**ICC 3776**	Iran	Desi	*Cicer arietinum*
**ICC 3946A**	Iran	Desi	*Cicer arietinum*
**ICC 3946B**	Iran	Desi	*Cicer arietinum*
**ICC 4093**	Iran	Desi	*Cicer arietinum*
**ICC 4182**	Iran	Desi	*Cicer arietinum*
**ICC 4363**	Iran	Desi	*Cicer arietinum*
**ICC 440**	India	Desi	*Cicer arietinum*
**ICC 4418**	Iran	Desi	*Cicer arietinum*
**ICC 4463**	Iran	Desi	*Cicer arietinum*
**ICC 4495**	Turkey	Desi	*Cicer arietinum*
**ICC 4533**	India	Desi	*Cicer arietinum*
**ICC 456**	India	Desi	*Cicer arietinum*
**ICC 4567**	India	Desi	*Cicer arietinum*
**ICC 4593**	India	Desi	*Cicer arietinum*
**ICC 4639**	India	Desi	*Cicer arietinum*
**ICC 4657**	India	Desi	*Cicer arietinum*
**ICC 4814**	Iran	Desi	*Cicer arietinum*
**ICC 4841**	Morocco	Kabuli	*Cicer arietinum*
**ICC 4872**	India	Pea-shaped	*Cicer arietinum*
**ICC 4918**	India	Desi	*Cicer arietinum*
**ICC 4948**	India	Desi	*Cicer arietinum*
**ICC 4958**	India	Desi	*Cicer arietinum*
**ICC 4973**	India	Desi	*Cicer arietinum*
**ICC 4991**	India	Desi	*Cicer arietinum*
**ICC 506**	India	Desi	*Cicer arietinum*
**ICC 5135**	India	Desi	*Cicer arietinum*
**ICC 5221**	India	Desi	*Cicer arietinum*
**ICC 5337**	India	Kabuli	*Cicer arietinum*
**ICC 5383**	India	Desi	*Cicer arietinum*
**ICC 5434**	India	Desi	*Cicer arietinum*
**ICC 5504**	Mexico	Desi	*Cicer arietinum*
**ICC 5613**	India	Desi	*Cicer arietinum*
**ICC 5639**	India	Desi	*Cicer arietinum*
**ICC 5845**	India	Desi	*Cicer arietinum*
**ICC 5878**	India	Desi	*Cicer arietinum*
**ICC 5879**	India	Pea-shaped	*Cicer arietinum*
**ICC 6263**	USSR	Kabuli	*Cicer arietinum*
**ICC 6279**	India	Desi	*Cicer arietinum*

**ICC 6293**	Italy	Desi	*Cicer arietinum*
**ICC 6294**	Iran	Desi	*Cicer arietinum*
**ICC 6306**	USSR	Desi	*Cicer arietinum*
**ICC 637**	India	Desi	*Cicer arietinum*
**ICC 6537**	Iran	Desi	*Cicer arietinum*
**ICC 6571**	Iran	Desi	*Cicer arietinum*
**ICC 6579**	Iran	Desi	*Cicer arietinum*
**ICC 67**	India	Desi	*Cicer arietinum*
**ICC 6802**	Iran	Desi	*Cicer arietinum*
**ICC 6811**	Iran	Desi	*Cicer arietinum*
**ICC 6816**	Iran	Desi	*Cicer arietinum*
**ICC 6874**	Iran	Desi	*Cicer arietinum*
**ICC 6875**	Iran	Desi	*Cicer arietinum*
**ICC 6877**	Iran	Desi	*Cicer arietinum*
**ICC 7052**	Iran	Desi	*Cicer arietinum*
**ICC 708**	India	Desi	*Cicer arietinum*
**ICC 7150**	Turkey	Desi	*Cicer arietinum*
**ICC 7184**	Turkey	Desi	*Cicer arietinum*
**ICC 7255**	India	Kabuli	*Cicer arietinum*
**ICC 7272**	Algeria	Kabuli	*Cicer arietinum*
**ICC 7305**	Afghanistan	Desi	*Cicer arietinum*
**ICC 7308**	Peru	Kabuli	*Cicer arietinum*
**ICC 7315**	Iran	Kabuli	*Cicer arietinum*
**ICC 7323**	USSR	Pea-shaped	*Cicer arietinum*
**ICC 7413**	India	Pea-shaped	*Cicer arietinum*
**ICC 7441**	India	Desi	*Cicer arietinum*
**ICC 7554**	Iran	Desi	*Cicer arietinum*
**ICC 7571**	Israel	Kabuli	*Cicer arietinum*
**ICC 762**	India	Desi	*Cicer arietinum*
**ICC 7668**	USSR	Kabuli	*Cicer arietinum*
**ICC 7819**	Iran	Desi	*Cicer arietinum*
**ICC 7867**	Iran	Desi	*Cicer arietinum*
**ICC 791**	India	Desi	*Cicer arietinum*
**ICC 8151**	USA	Kabuli	*Cicer arietinum*
**ICC 8195**	Pakistan	Desi	*Cicer arietinum*
**ICC 8200**	Iran	Desi	*Cicer arietinum*
**ICC 8261**	Turkey	Kabuli	*Cicer arietinum*
**ICC 8318**	India	Desi	*Cicer arietinum*
**ICC 8350**	India	Pea-shaped	*Cicer arietinum*
**ICC 8384**	India	Desi	*Cicer arietinum*
**ICC 8515**	Greece	Desi	*Cicer arietinum*
**ICC 8522**	Italy	Desi	*Cicer arietinum*
**ICC 8621**	Ethiopia	Desi	*Cicer arietinum*
**ICC 867**	India	Desi	*Cicer arietinum*
**ICC 8718**	Afghanistan	Desi	*Cicer arietinum*
**ICC 8740**	Afghanistan	Kabuli	*Cicer arietinum*
**ICC 8752**	Afghanistan	Kabuli	*Cicer arietinum*
**ICC 8855**	Afghanistan	Kabuli	*Cicer arietinum*
**ICC 8950**	India	Desi	*Cicer arietinum*
**ICC 9002**	Iran	Desi	*Cicer arietinum*
**ICC 9137**	Iran	Kabuli	*Cicer arietinum*
**ICC 9402**	Iran	Kabuli	*Cicer arietinum*
**ICC 9434**	Iran	Kabuli	*Cicer arietinum*
**ICC 95**	India	Desi	*Cicer arietinum*
**ICC 9586**	India	Desi	*Cicer arietinum*
**ICC 9590**	Egypt	Desi	*Cicer arietinum*
**ICC 9636**	Afghanistan	Desi	*Cicer arietinum*
**ICC 9643**	Afghanistan	Desi	*Cicer arietinum*
**ICC 9712**	Afghanistan	Desi	*Cicer arietinum*
**ICC 9755**	Afghanistan	Desi	*Cicer arietinum*
**ICC 9848**	Afghanistan	Pea-shaped	*Cicer arietinum*
**ICC 9862**	Afghanistan	Pea-shaped	*Cicer arietinum*
**ICC 9872**	Afghanistan	Kabuli	*Cicer arietinum*
**ICC 9895**	Afghanistan	Pea-shaped	*Cicer arietinum*
**ICCV 95311**	India	Kabuli	*Cicer arietinum*
**ILWC 235**	India	Desi	*Cicer echinospermum*
**ILWC 245**	India	Desi	*Cicer echinospermum*
**Neelam**	Australia	Desi	*Cicer arietinum*

**PBA Boundary**	Australia	Desi	*Cicer arietinum*
**PBA Slasher**	Australia	Desi	*Cicer arietinum*
**PBA Striker**	Australia	Desi	*Cicer arietinum*
**WACPE2160**	Australia	Desi	*Cicer arietinum*

USSR, Union of Soviet Socialist Republics.

Table S2. Descriptive statistics by seed types (desi, kabuli, and pea-shaped) of 33 measured traits (30 root traits, and three shoot traits) in 270 chickpea genotypes

Trait	Minimum			Maximum			Mean			Median			Std. Deviation			CV*		
	Desi	Kabuli	PS	Desi	Kabuli	PS	Desi	Kabuli	PS	Desi	Kabuli	PS	Desi	Kabuli	PS	Desi	Kabuli	PS
**TRL_z1**	17.0	33.0	37.3	80.0	78.7	70.0	55.8	55.4	55.9	55.7	55.0	56.8	11.9	11.1	10.9	0.21	0.20	0.19
**TRL_z2**	2.67	6.00	9.00	36.3	34.0	23.7	16.5	16.2	15.9	16.0	16.0	16.8	6.16	5.31	5.61	**0.37**	**0.33**	**0.35**
**TRL**	38.3	40.5	48.3	105.0	95.3	88.5	72.2	71.6	71.9	72.0	72.0	74.2	12.1	12.5	14.7	0.17	0.17	0.20
**RL**	305	539	546	3824	2938	1271	1163	1249	1019	1063	1132	1197	450	481	295	**0.39**	**0.39**	0.29
**BL**	267	480	497	3765	2844	1190	1091	1177	947	997	1062	1126	445	475	285	**0.41**	**0.40**	**0.30**
**BN**	23.1	65.8	45.2	764	604	236	186	202	154	156	164	176	103	114	70.9	**0.55**	**0.57**	**0.46**
**ABL**	4.11	4.62	4.90	14.0	12.24	10.64	6.92	6.95	7.02	6.78	6.91	6.46	1.51	1.60	1.87	0.22	0.23	0.27
**RA**	94.8	164	137	1027	704	355	313	350	271	290	335	299	123	125	78.1	**0.39**	**0.36**	0.29
**RV**	2.36	3.52	2.53	22.0	13.4	8.29	6.77	7.88	5.79	6.19	7.80	6.00	2.75	2.70	1.77	**0.41**	**0.34**	**0.31**
**RD**	0.68	0.77	0.72	1.02	1.07	0.94	0.86	0.90	0.85	0.86	0.89	0.88	0.06	0.06	0.07	0.06	0.07	0.09
**SRL**	32.5	33.4	38.0	264	184.8	70.3	67.7	59.8	53.7	61.1	53.4	53.3	28.1	24.7	9.56	**0.41**	**0.41**	0.18
**BLR_tap**	6.62	7.78	9.80	77.8	39.9	19.8	15.4	16.9	13.4	14.2	16.1	13.4	6.81	6.28	3.28	**0.44**	**0.37**	0.25
**BD**	0.57	0.95	1.04	15.73	7.62	3.34	2.57	2.84	2.12	2.27	2.57	2.29	1.55	1.52	0.87	**0.60**	**0.53**	**0.41**
**BI**	0.08	0.10	0.10	0.24	0.22	0.20	0.16	0.16	0.15	0.15	0.15	0.16	0.03	0.03	0.03	0.19	0.20	0.22
**RTD**	1.27	1.60	2.71	5.87	9.87	6.09	2.98	3.21	3.76	3.04	3.09	3.37	0.69	1.09	1.24	0.23	**0.34**	**0.33**
**RL_top**	140	250	216	727	923	611	427	490	441	422	454	445	110	152	144	0.26	**0.31**	**0.33**
**BL_top**	120	230	196	707	903	591	407	470	421	402	434	425	110	152	144	0.27	**0.32**	**0.34**
**RD_top**	0.77	0.79	0.80	1.04	1.09	0.98	0.91	0.96	0.90	0.91	0.95	0.91	0.05	0.06	0.07	0.05	0.06	0.08
**RL_s2**	71.6	191	117	868	858	618	419	473	364	403	425	404	148	189	142	**0.35**	**0.40**	**0.39**
**RD_s2**	0.64	0.69	0.68	0.98	1.00	0.89	0.81	0.84	0.79	0.81	0.84	0.82	0.06	0.06	0.08	0.07	0.07	0.10
**RL_s3**	31.8	21.1	45.4	827	620	412	266	276	211	246	264	183	140	136	122	**0.53**	**0.49**	**0.57**
**RD_s3**	0.64	0.62	0.69	1.04	1.05	0.99	0.85	0.89	0.85	0.86	0.90	0.85	0.07	0.09	0.10	0.09	0.10	0.12
**RL_sub**	144	267	162	1575	1326	870	685	749	575	644	696	624	258	295	225	**0.38**	**0.39**	**0.39**
**BL_sub**	126	229	134	1497	1263	801	633	697	524	595	640	562	251	288	216	**0.40**	**0.41**	**0.41**
**RD_sub**	0.64	0.70	0.68	1.00	1.03	0.94	0.83	0.87	0.82	0.83	0.87	0.84	0.06	0.07	0.09	0.08	0.08	0.10
**RLR_top/sub**	0.23	0.38	0.46	2.00	2.20	1.48	0.75	0.83	0.99	0.68	0.71	0.94	0.30	0.40	0.34	**0.40**	**0.48**	**0.34**
**BLR_top/sub**	0.23	0.38	0.48	2.25	2.63	1.59	0.79	0.88	1.07	0.71	0.73	0.98	0.34	0.47	0.39	**0.43**	**0.53**	**0.37**
**RGR**	1.10	1.16	1.38	3.00	2.72	2.53	2.06	2.05	2.05	2.06	2.06	2.12	0.35	0.36	0.42	0.17	0.17	0.20

**RM**	69.3	102	105	353	370	325	189	231	207	187	216	207	56.7	69.2	68.5	**0.30**	**0.30**	**0.33**
**SM**	119	190	136	1006	894	463	308	419	295	280	388	300	127	167	106	**0.41**	**0.40**	**0.36**
**RMR**	0.19	0.26	0.490	1.23	1.55	0.884	0.72	0.61	0.73	0.70	0.59	0.78	0.38	0.23	0.12	**0.52**	**0.37**	0.16
**SH**	8.23	11.1	9.83	30.0	29.7	25.1	16.3	18.7	16.7	16.4	19.0	17.4	4.23	3.55	5.55	0.26	0.19	**0.33**
**LBN**	10.0	11.3	11.3	55.7	16.0	14.0	13.7	13.4	13.0	13.3	13.3	13.0	4.12	0.99	0.73	**0.30**	0.07	0.06

Traits with CVs (coefficients of variation) ≥0.30 appear in bold type. PS, Pea-shaped.

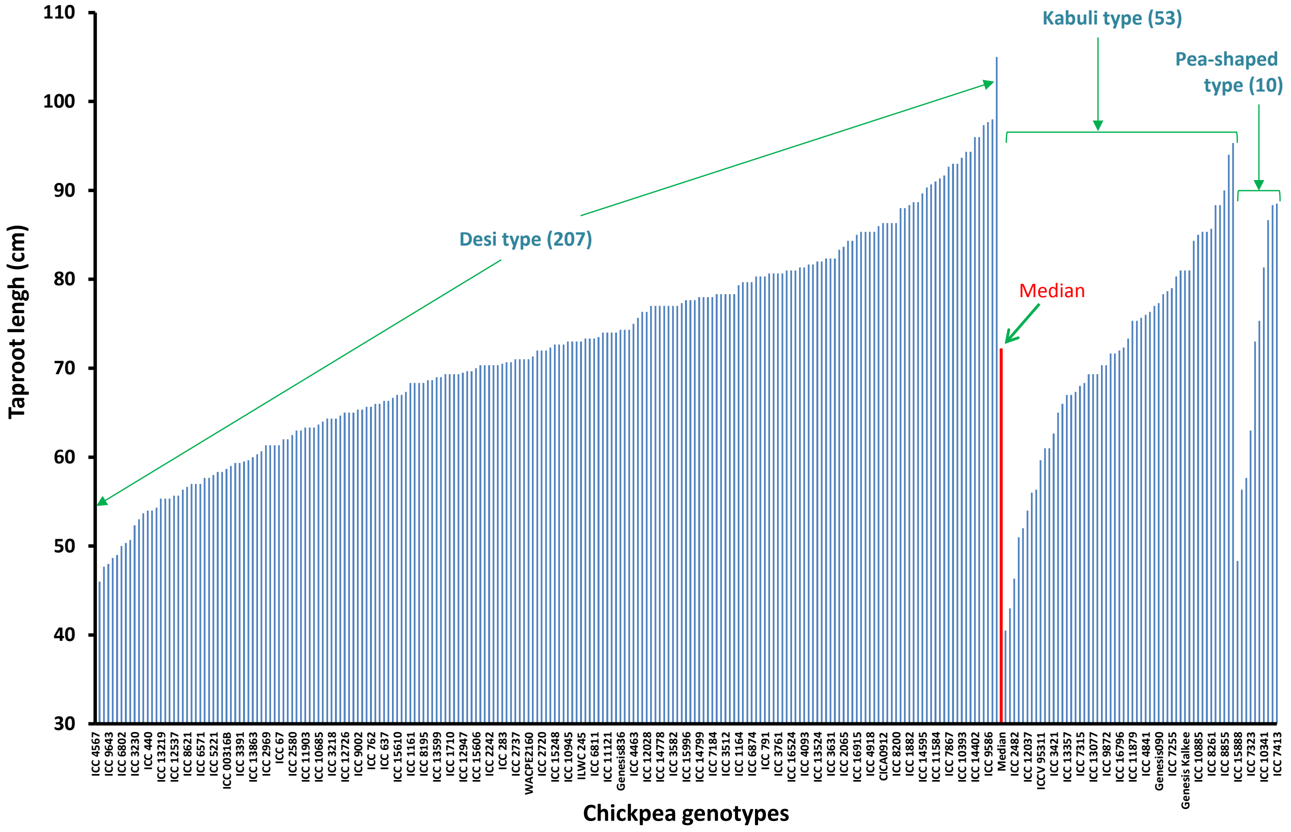

Fig. S1. Genotypic variation in taproot length among 270 chickpea genotypes plotted by seed types (desi, kabuli, and pea-shaped).

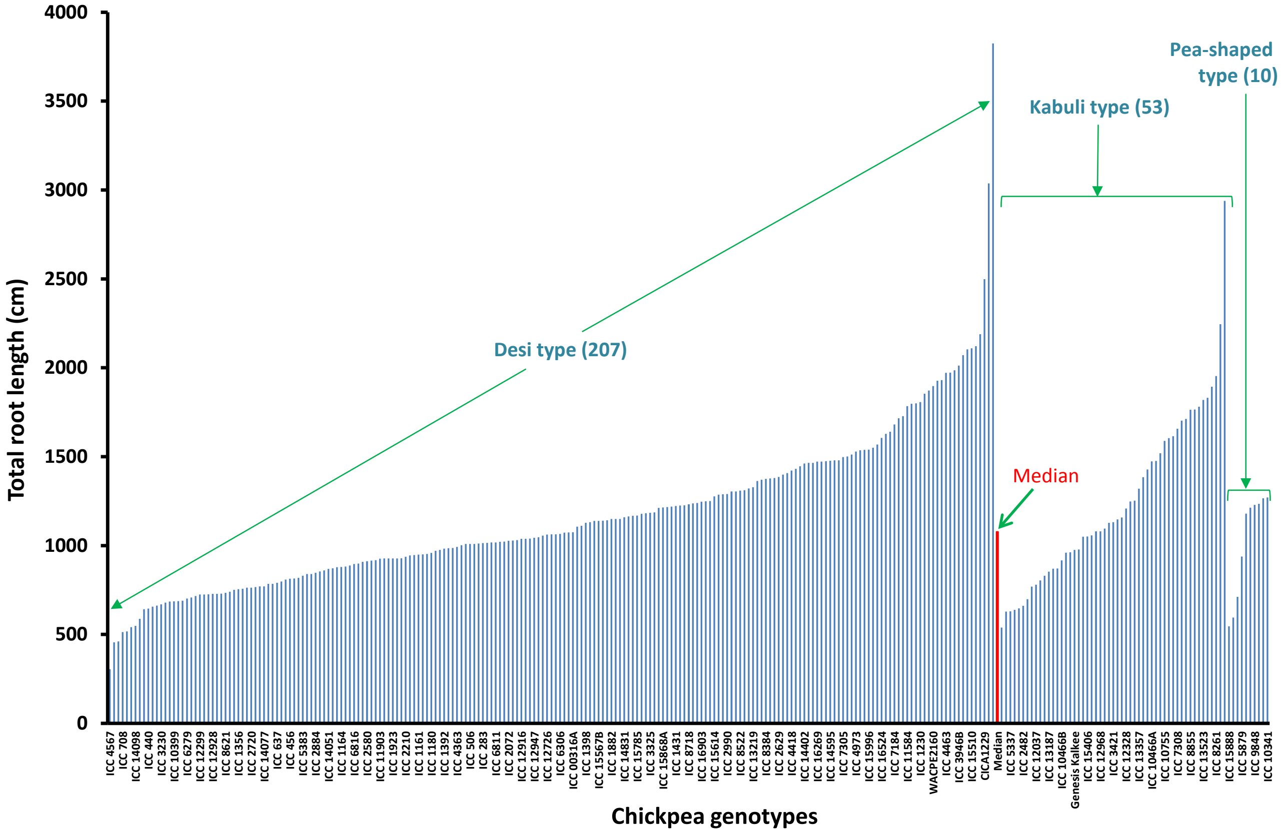

Fig. S2. Genotypic variation in total root length among 270 chickpea genotypes plotted by seed types (desi, kabuli, and pea-shaped).
